# Pan-Immune Inflammation Value as a Novel Comprehensive Predictor of In-Hospital Mortality in Patients with Severe Burns: A Single-Center Retrospective Analysis

**DOI:** 10.3390/medicina61091705

**Published:** 2025-09-19

**Authors:** Hilmi Anil Dincer, Sara Koci, Omer Cennet, Ali Konan

**Affiliations:** General Surgery Department, Hacettepe University Faculty of Medicine, Ankara 06230, Turkey; sara.koci@hacettepe.edu.tr (S.K.); omer.cennet@hacettepe.edu.tr (O.C.); akonan@hacettepe.edu.tr (A.K.)

**Keywords:** severe burn, Pan-immune inflammation value, burn mortality, inflammation indexes

## Abstract

*Background and Objectives*: Despite the advances in the treatment, severe burns with total burn surface area ≥ 20% are still a major cause of mortality worldwide. Pan-immune inflammation value (PIV) is a novel and promising biomarker to predict prognosis and mortality in various diseases. The aim of this study was to evaluate the utility of PIV to predict in-hospital mortality of patients with severe burn. *Materials and Methods*: This retrospective cross-sectional study included ≥18 years old patients with severe burn who were admitted to hospital within 12–24 h after the burn injury between January 2007 and August 2024. The demographics, clinical and laboratory characteristics of patients were recorded from electronic hospital records. Pan-immune inflammation value was calculated as neutrophil counts x monocyte count x platelet counts divided by lymphocyte counts. The receiver operating characteristic (ROC) analysis was conducted to determine the predictive value of PIV for mortality. *Results*: A total of 100 patients (median age 41 (26.3–55) years; 79% male) were included in the study of whom 23 were non-survivors. The PIV was significantly higher in non-survivors when compared to survivors (*p* = 0.009). The ideal cut-off of PIV was 1185, with a sensitivity of 69.6% and a specificity of 66.2%. The multivariate analysis showed that high PIV along with inhalation injury, and the need for surgery were predictors of in-hospital mortality. *Conclusions*: This study is the first to demonstrate that the novel, comprehensive index, PIV, is a reliable immuno-inflammatory marker predicting in-hospital mortality in patients with severe burn.

## 1. Introduction

Burn injuries rank as the fourth most prevalent type of injury worldwide with an increased risk of long-term morbidity and significant mortality [1]. According to the World Health Organization, burns are responsible for at least 265,000 deaths annually [2]. The main risk factors for increased mortality in burn patients are age, sex, total body surface area affected by the burn (TBSA), type of burn, presence of inhalation injury, comorbidities, and the nutritional status [3,4].

Extensive burns are among the most frequent severe injuries, and even with recent significant advancements in burn care, patients with severe burns still face higher mortality rates and longer hospital stays leading to increased medical expenses and economic burden [5]. Therefore, to improve the outcomes of patients with severe burn, it is crucial to assess the disease progression and mortality risk at the time of hospital admission.

Overactivation of immune system is usually induced after burns, especially after severe burns, an extensive and long-term immune response activation is seen both locally in the burn wound and systemically in the peripheral blood [6,7]. The animal studies showed that both innate and adaptive immune systems are triggered in response to the burn injury, resulting in an increase in the number of neutrophils, monocytes and thrombocytes in the peripheral blood shortly after the burn with a decrease in lymphocyte counts during the first two weeks of the injury [6]. As the severity of the burn increases, such as in cases of extensive burn injury, the hyperactivation of the immune system may last for months which leads to the persistence of the systemic inflammatory response syndrome that in turn results in multi-organ damage, long-term morbidity, and even death [1].

Burn injuries cause profound alterations in the complete blood count (CBC), reflecting both the inflammatory and hemostatic response to tissue damage. In patients with severe burns, neutrophil and monocyte counts in peripheral blood are elevated, whereas lymphocyte counts remain relatively unchanged, indicating that the immune system stays in a prolonged proinflammatory state rather than transitioning toward resolution [8]. In addition to leukocyte alterations, platelet kinetics show a characteristic evolution after burn injury. Several studies have demonstrated an early transient thrombocytopenia, with platelet counts reaching a nadir around days 2–5 post-burn, followed by a rebound thrombocytosis between days 11 and 17 [2,3]. Recently, Martín Angulo et al. systematically analyzed the evolution of complete blood count and derived indices in burn patients and confirmed that platelet dynamics, along with indices incorporating platelet counts, provide independent prognostic information [4].

Immune-inflammatory biomarkers such as neutrophils, monocytes, lymphocytes, and platelets which are easily and readily obtained from the complete blood count have been widely used in recent studies to show the immune and inflammatory status of patients with malignancies and other inflammatory diseases [5,6,7]. Since the neutrophil/lymphocyte ratio (NLR) was shown in 2001 to be more accurate in predicting the prognosis and survival of patients than the immune cell counts alone, numerous studies focused on the utility of other indices such as lymphocyte/platelet ratio (LPR), monocyte to lymphocyte ratio (MLR), systemic immune inflammatory index (SII) and systemic inflammation response index (SIRI) in various diseases [9]. With the growing evidence that these markers are reliable predictors of survival in critically ill patients, studies were also conducted in burn patients to assess early mortality risk and previous studies showed that NLR, LPR, MLR and SII were reliable predictors of prognosis and mortality in patients with extensive burns [10,11,12,13,14].

Pan-immune inflammation value (PIV) [(neutrophil count ×  platelet count  ×  monocyte count)/lymphocyte count] is a novel immune-inflammatory marker which was first demonstrated to predict survival better than other inflammatory biomarkers in patients with metastatic colorectal cancer [15]. As PIV integrates all four of the previously mentioned immune cells, it has a potential to accurately depict the systemic inflammation and immunity which has been shown as a promising biomarker to identify the prognosis of patients with malignancies and other inflammation related diseases [16]. Given the complex pathophysiology of severe burns, the PIV, may offer greater utility than other biomarkers for predicting mortality risk in extensive buns. However, to the best of our knowledge, no studies have been conducted with extensive burn patients on this topic.

The objective of this study was to assess the predictive value of PIV at the time of hospital admission for in-hospital mortality in patients with extensive burn. The secondary endpoint of the study was to evaluate the association between PIV and other burn severity scores.

## 2. Materials and Methods

### 2.1. Study Design and Patient Selection

This retrospective cross-sectional study included ≥18 years old patients with extensive burn; defined as TBSA ≥ 20% in adults [8] who were admitted to our burn center at Hacettepe University Faculty of Medicine, Ankara, Turkey between January 2007 and August 2024. Inclusion criteria of the study were as follows: (i) patients classified as having extensive burn when they had TBSA ≥ 20% with ≥2nd degree burn [8]; (ii) admission to hospital within the 12–24 h of the burn injury. The exclusion criteria included (i) patients under 18 years old; (ii) patients with a burn degree < 20% TBSA and/or 1st degree burn; (iii) pregnant or lactating patient; (iv) patients with incomplete data; (v) patients who had concomitant lymphoproliferative or solid malignancy, and/or had chemo/radiotherapy at the time of the burn injury and (vi) patients taking any immunosuppressive or corticosteroid treatments. These patient groups were excluded to avoid confounding effects of underlying diseases or medications that could alter immune cell counts and potentially affect study outcomes.

Patients’ data regarding age, sex, burn injury type, TBSA, presence of inhalation injury, need for surgery, comorbidities, and in-hospital mortality were retrieved from hospital records retrospectively.

### 2.2. Laboratory Data

The laboratory data included CBC with differentials performed, serum albumin, creatinine, and liver transaminases at the time of hospital admission (day 1) which were retrieved from electronic hospital records. The following indices were calculated from differentials of CBC: NLR, LPR, MLR, SII calculated as (neutrophil counts × platelet counts)/lymphocyte counts, and SIRI calculated as (neutrophil counts × monocyte counts)/lymphocyte counts. Pan-immune inflammation value was calculated as neutrophil counts × monocyte count × platelet counts divided by lymphocyte counts. The hemoglobin (Hb) value, red cell distribution width (RDW), mean platelet volume (MPV) and mean corpuscular volume (MCV) were also recorded from complete blood count.

### 2.3. Burn Severity Indices

The following mortality predicting indices were calculated for each patient on day one.

i. Abbreviated Burn Severity Index (ABSI) was first created by Tobiasen et al. in 1982 and is still a widely used scoring system to predict the mortality after burns [9]. This scoring system consists of five parameters as age, sex, TBSA, full thickness burn (3rd degree burn), and inhalation injury. The parameters are scored accordingly, the total score ranges from 2 to 13, and each score corresponds to a specific probability of survival.
ii. The Revised Baux Score (rBaux) was first proposed by Osler et al. in 2010, and is calculated as: Age + TBSA + 17 * (Inhalation Injury, 1 = yes, 0 = no) [10]. This is an easily applied scoring system with a high predictive value of mortality.
iii. Belgian Outcome in Burn Injury (BOBI) was first developed and validated by The Belgian Outcome in Burn Injury Study Group which includes age (0–3 points: 0, <50 years; 1, 50–64 years; 2, 65–79 years; 3, ≥80 years), TBSA (0–4 points: 0, <20%; 1, 20–39%; 2, 40–59%; 3, 60–79%; 4, ≥80%), and presence of inhalation injury (3 points). The total score ranges from 0 to 10 where each total score corresponds a mortality percentage [11].
iv. Cape Town Burn Score (CTBS) was first proposed in 1998 by Godwin et al. and is calculated as: Age + %TBSA burn + (20 × inhalation injury grade) [12].
v. Sepsis-related Organ Failure Assessment (SOFA) Score is widely used score to assess the organ dysfunction or failure of patients in intensive care unit which evaluates six organ systems. Each organ system is scored 0 to 4 and total score ranges from 0 to 24. The higher scores are associated with higher mortality rate [13].

### 2.4. Statistical Analysis

The data analysis was performed using IBM SPSS (Statistical Package for the Social Sciences) version 27 (Armonk, NY, USA). The normality of the distribution of variables was examined using both visual (histogram and probability graphs) and analytical methods (Kolmogorov–Smirnov/Shapiro–Wilk tests). Descriptive analyses were presented as mean and standard deviation for normally distributed numerical variables; median and interquartile range for non-normally distributed numerical variables; and frequency tables for ordinal and categorical variables. Comparisons between groups were performed by using Student’s T-Test was used for normally distributed numerical variables, the Mann–Whitney U test for non-normally distributed numerical variables, and the Chi-square or Fisher’s exact test, as appropriate, for categorical variables. The Spearman test was used to conduct a correlation analysis between PIV and other indices. The receiver operating characteristic (ROC) analysis was conducted to determine the predictive value of PIV for mortality. When significant cut-off values were present, positive likelihood ratios and Youden indices for these values were calculated. ROC analysis was performed, and the area under the curve (AUC) was calculated for ABSI, rBaux, BOBI, CTBS, SOFA, and NLR to evaluate their ability to predict mortality. Sensitivity, specificity, positive predictive value, and negative predictive value were also assessed. The AUC values between 0.6–0.7, 0.7–0.8, 0.8–0.9, and >0.9 were interpreted as indicating acceptable, fair, good, and excellent discriminatory performance, respectively [14]. Multivariate analysis was conducted to determine the risk factors for mortality. In univariate analyses, statistically significant parameters were included in the model, and logistic regression analysis was applied. In correlation analysis, when the correlation coefficient between two variables was above 0.6, the one with a higher *p*-value or lower clinical significance was excluded from the analysis. Model fit was evaluated with the Hosmer-Lemeshow test. Results were considered statistically significant for *p* < 0.05.

## 3. Results

A total of 100 patients with extensive burn were included in this study. The median age of patients was 41 (26.3–55) years old, and the majority of patients were male (*n* = 79). The main burn injury was flaming injury (69%) followed by scald (22%), electrical (8%) and chemical (1%) burns. The median TBSA of the whole group was 27.5% (IQR:20–50), and 27% of the patients had concurrent inhalation injury. The in-hospital mortality prediction scores of all patients were as follows: rBaux score 76 (IQR: 59.3–103.8), ABSI 6 (IQR: 5–9), BOBI 2 (IQR: 1–4), CTBS 4 (IQR: 3–5), and SOFA score 1 (IQR: 0–3). The clinical and demographic features of patients with extensive burn were summarized in Table 1.

A total of 23 patient out of 100 patients were non-survivors. There was statistically no significance between age, sex and the presence of comorbidities between survivors and non-survivors. However, non-survivor group had significantly higher TBSA, ABSI, rBaux, BOBI, and CTBS scores compared to survivor group (*p* < 0.001, *p* < 0.001, *p* < 0.001, *p* = 0.014, andp = 0.021, respectively). On the other hand, the SOFA score of survivors (0 [0–3]) and non-survivors (1 [0–4]) did not differ significantly (*p* = 0.10). There were no significant differences between Hb, lymphocyte, monocyte, and platelet counts between survivor and non-survivor groups as well as the MCV, MPV and RDW values did not differ between groups. On the other hand, neutrophil counts were significantly higher, and serum albumin levels were significantly lower in non-survivors in comparison to survivors (*p* < 0.001 and *p* < 0.001, respectively). The ratio of RDW to serum albumin was significantly higher in non-survivors (6.4 [5.7–5.2]) compared to the survivors’ group (3.9 [1.16–4.48], *p* < 0.001).

Moreover, the SII, SIRI and PIV were statistically significantly higher in non-survivors when compared to survivors (*p* < 0.001, *p* < 0.001, *p* = 0.009, respectively).

The pan-immune inflammatory index showed statistically significant positive correlations with SII, SIRI, NLR, LPR, MLR and RDW/albumin ratios (Table 2). Even though PIV was significantly correlated with ABSI (r_s_ = 0.229, *p* = 0.022), a significant association between rBaux, BOBI, CTBS, SOFA scores and PIV was not observed (r_s_ = 0.169, *p* = 0.093, r_s_ = 0.108, *p* = 0.29, r_s_ = 0.068, *p* = 0.50, r_s_ = 0.117, *p* = 0.25, respectively) (Table 2).

By using ROC analysis to determine the predictive value of PIV for survival, the ideal cut-off was 1185, with a sensitivity of 69.6%, specificity of 66.2%, positive likelihood ratio of 2.06, and a Youden index of 0.36 (AUC: 0.679, 95% CI: 0.552–0.806, *p* = 0.009). When this cut-off was used, 69.9% of non-survivors compared to 33.8% of survivors had PIV ≥ 1185 (*p* = 0.002).

**Figure 1:**
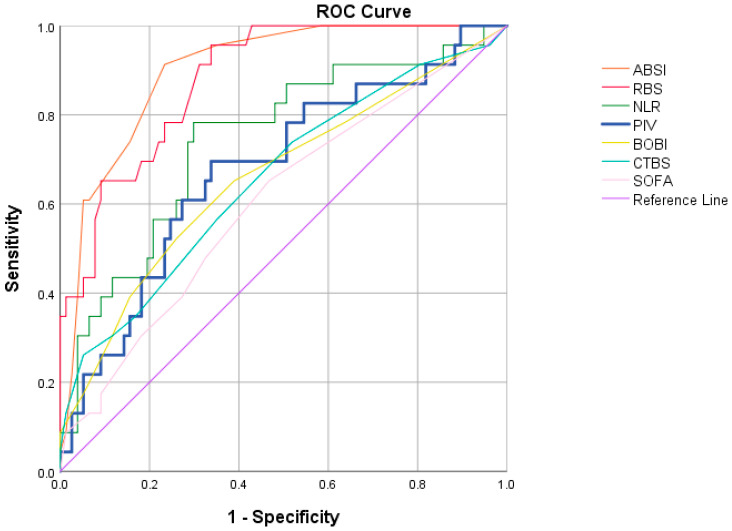
The Receiver operating characteristic analysis of the mortality prediction scores. ABSI; Abbreviated Burn Severity Index, BOBI; Belgian Outcome in Burn Injury, CTBS; Cape Town Burn Score, NLR; neutrophil/lymphocyte ratio, PIV; Pan-immune inflammation value, RBS; revised Baux score, SOFA; Sepsis-related Organ Failure Assessment.

The ROC curve analysis demonstrated that ABSI had the highest predictive accuracy with an AUC of 0.90, followed by r-Baux (0.83), which also showed strong discriminatory ability. The neutrophil-to-lymphocyte ratio ranked third with a fair predictive value (0.74). In contrast, PIV (0.68), BOBI (0.66), CTBS (0.66), and SOFA (0.61) performed less effectively, indicating only limited discriminatory capacity (Figure 1). Overall, ABSI emerged as the most reliable predictor of mortality, whereas SOFA showed the weakest performance. The detailed sensitivity, specificity, positive predictive value, negative predictive value, and AUC results for each scoring system were summarized in Table 3.

We analyzed the independent variables that predicted mortality further using logistic regression models. In the multivariate analysis, the presence of inhalation injury, the need for surgery and high pan-immune inflammation value were found to be predictors of in-hospital mortality in patients with extensive burn (Table 4).

## 4. Discussion

In this study, we aimed to evaluate the prognostic value of the Pan-Immune-Inflammation Value in predicting in-hospital mortality among patients with severe burn injury. In this cohort, which included individuals admitted to the hospital with extensive burns, PIV at admission was significantly higher among non-survivors than survivors. Also, we showed that PIV was significantly correlated with burn severity index ABSI and the other immune-inflammatory markers. While limited studies in the literature have revealed the relationship between NLR, PLR and burn prognosis, our study is, to the best of our knowledge, the first to demonstrate the association of PIV-the novel comprehensive index incorporating all four immune cells, with in-hospital mortality in patients with severe burn.

Severe burn injury has been defined as TBSA ≥ 20% in adult patients and was shown to have a high mortality rate up to 18% despite the advances in the treatment and burn care [8,15]. Studies demonstrated that age, TBSA, type of burn, inhalation injury, comorbidities and nutritional status are risk factors for increased mortality [16]. In this current study, the presence of inhalation injury, the need for surgery and high PIV were shown to be predictors of in-hospital mortality in patients with extensive burn. In our cohort, though, as comorbidities did not differ significantly between survivors and non-survivors and were therefore not included in the uni- and multivariate analyses. However, it should be noted that only a limited number of comorbidities were assessed due to the retrospective design of the study, which may have underestimated their potential effect on outcomes.

Accurate early prediction of outcomes and prognosis in patients with major burns enables physicians to establish effective management strategies and treatment goals, ensuring optimal care while minimizing long-term mortality, morbidity, and economic impact. Since 1961, indices such as Baux, rBaux, ABSI, BOBI, and CTBS to determine mortality in burn patients have been proposed with varying specificity and sensitivity; however, most of these indices are complex to calculate and non-practical to use in daily practice. Thus, the availability of markers that are reliable, simple to calculate, and easily obtained from routine blood tests would be valuable to assess the prognosis of patients upon hospitalization.

Severe burn injury causes an augmented and prolonged inflammatory response which may last for months resulting in systemic inflammatory response syndrome, hypermetabolic state and damage [17]. With the activation of innate immune system, neutrophils and macrophages are the first immune cells to be released which in turn enhance the inflammatory reaction via secretion of various proinflammatory cytokines. Studies showed that in burn patients, the number of neutrophils and monocytes increased in the peripheral blood while the lymphocyte counts did not change [18]. The platelet count was shown to decrease after the burn injury and remained lower in non-survivors compared to survivors [2]. In recent years, the use of certain indices derived from CBC have been expanded in many therapeutic settings. Even though each of the immune cells play an important role in inflammation, using them alone is not sufficient to detect inflammation. Therefore, with the demonstration of the relationship between NLR and systemic inflammation, novel indices that incorporate other immune cells have been developed to more precisely predict the prognosis of numerous diseases [19]. The systemic immune inflammation index and SIRI which included three of immune cells were found to be more accurate in predicting poor survival in patients with malignancies compared to indices that incorporate only two immune cells [20,21]. More recently, in 2020, the PIV was described as combining all of the four immune cells and shown superior to SII in predicting overall survival in metastatic colorectal cancer [22].

Severe burns were shown to cause changes in complete blood counts and trending CBC indices for survivors and non-survivors could provide valuable references to help clinicians predict the clinical outcome of severe burn patients [2]. The NLR, PLR, and MLR have been shown to be related with systemic inflammation, thus their utility in predicting prognosis in various cancer types, autoimmune diseases, coronary artery disease and other inflammatory diseases have been widely investigated [23,24,25]. Several studies have also been conducted in burn injuries to predict the prognosis and survival [26,27]. In our study, we showed that at the time of hospital admission, non-survivor group had significantly higher neutrophil count when compared to survivor group, whereas the lymphocyte, monocyte and platelet counts did not significantly differ between the two groups. However, in our cohort, although the difference was not statistically significant, non-survivors demonstrated a trend toward lower platelet counts compared to survivors. This observation was in line with previous studies reporting early thrombocytopenia in severe burns. Additionally, we demonstrated that NLR and MLR were statistically significantly higher in the non-survivors, even though LPR was not significantly different between survivors and non-survivors but tended to be higher in non-survivors. Similarly to our results, Angulo et al. showed that deceased patients had higher neutrophil count at day 1 than the survived patients (*p* < 0.001) but lymphocyte and platelet counts did not differ between the groups at day 1 [4]. They also showed that NLR at the time of admission and PLR at day 3 were higher in deceased patients [4]. In a recently published meta-analysis which included nine studies with a total of 1837 burn patients of whom 1526 survived, the overall mean difference revealed a significant increase in NLR of 5.06 (95% CI 3.42, 6.68) for the non-survivor group compared to the survivor group, *p* ≤ 0.001 [28]. In a study conducted with a total of 100 severely burned patients (TBSA >50%), MLR was significantly higher in non-survivors during the first two days of the injury, but on the 3rd, 5th, 6th, and 7th days, the values were lower in non-survivors compared to survivors. The MLR value on the 6th day after the injury was found as an independent predictor of mortality [29].

As previously mentioned, neutrophils, along with macrophages, are the first line immune cells that are activated after the burn injury and infiltrate the injured area, which may explain the difference between neutrophil counts in survivor and non-survivor patients with major burn at the time of hospital admission. However, the number of lymphocytes, monocytes and platelets vary throughout the course of burn and since our study only focused on CBC at the time of admission, the lack of difference between the two groups in cell counts other than neutrophils may be attributed to this factor. Yet, our study, consistent with the previous studies [29,30,31], demonstrated that NLR and MLR at hospital admission were higher in deceased patients, suggesting that these parameters could be used to predict the prognosis of severely burned patients.

Previous studies showed that the combination of NLR, PLR and MLR had higher diagnostic and prognostic accuracy [23]. The novel composite indices such as SII, SIRI and the most recently described PIV integrate the three white blood cells subsets and/or platelets. They are thought to reflect the interaction between inflammation, immunity and thrombocytosis [32]. In this current study, we demonstrated that SII, SIRI and PIV were significantly higher in non-survivors in comparison to survivors. Also, PIV was significantly associated with burn severity indices and was shown to be a predictor of in-hospital mortality in multivariate logistic analysis. Similarly, in a newly published study in 2025 that involved 223 patients with facial burn, PIV was found significantly higher in patients with scar formation (*n* = 106) 7 days post-treatment compared to patients without scar formation (*n* = 117), (*p* < 0.001) which showed that increased PIV was strongly associated with an increased risk of scar formation [33]. In another study conducted with pediatric burn patients, higher SII was associated with longer hospital stay and having more full-thickness burn; however, the main limitation of this study, as stated by the authors, was the inclusion of small number of patients without major burn. The authors concluded that burns involving a TBSA > 20% trigger an acute systemic response, which may have a greater impact on changes in the calculated parameters. Therefore, the SII would provide more valuable information in major burns compared to minor burns [34]. Lately, there has been a growing interest in evaluating the significance of PIV in predicting prognosis in other immune-inflammatory diseases. In a recent study conducted with 11,331 septic patients, higher PIV was significantly associated with 28-day and 90-day of mortality in septic patients [35]. In another new study that included 542 patients diagnosed with ST-segment elevation myocardial infarction (STEMI), it was shown that PIV had a greater predictive value of the occurrence of major adverse cardiovascular event and the degree of coronary stenosis in hospitalized STEMI patients [36]. To the best of our knowledge, no previous study has evaluated the prognostic value of PIV in patients with extensive burns. Our findings suggest, for the first time, that PIV may serve as a reliable, affordable, and readily available comprehensive biomarker for predicting in-hospital mortality in this patient population. While further validation in larger, prospective cohorts is required, PIV could potentially be incorporated into routine admission assessments to aid early risk stratification and guide closer monitoring of high-risk patients. Moreover, PIV may complement existing severity scoring systems by providing additional insight into patients’ inflammatory status and overall prognosis, potentially supporting more individualized management strategies.

The findings of this study reinforce the superior predictive accuracy of ABSI, which demonstrated the highest (AUC: 0.90) followed by r-BAUX (AUC: 0.83) for in-hospital mortality prediction in burn patients. In a meta-analysis published in 2022 that included 98,610 burn patients, it was shown that r-Baux (AUC: 0.92) and ABSI (AUC: 0.89) scores had the highest discriminating ability in predicting mortality [37]. Similarly, in a recent study by Obed et al. where they compared the composite mortality prediction scores, ABSI (AUC: 0.904) and r-Baux (AUC: 0.900) had the highest values [38]. Although the AUC values for BOBI and CTBS were lower in our study compared to previous reports, their discriminative ability for predicting mortality remains, compatible with previous studies, inferior to that of ABSI and r-Baux [37,38]. SOFA (AUC 0.61), performed the weakest in our study (AUC: 0.61), consistent with prior studies that showed the AUC of SOFA as 0.79 (95% CI: 0.65–0.94) [37]. The neutrophil-to-lymphocyte ratio showed fair discriminatory ability (AUC 0.74), in line with recent studies recognizing its role as an accessible and cost-effective biomarker for mortality risk in burn patients [30]. Although PIV showed only modest discrimination in our cohort, it ranked after ABSI and r-Baux and outperformed BOBI, CTBS, and SOFA. As a novel index reflecting systemic inflammatory status, PIV may serve as a practical, early tool for mortality risk assessment due to its reliance on routine laboratory parameters. Nevertheless, further studies in larger cohorts are needed to validate its accuracy and to determine whether integrating PIV with established scores could enhance predictive performance.

Another interesting finding from the present study was a significantly higher RDW/albumin ratio in non-survivors (6.4 [5.7–5.2]), compared to survivors (3.9 [3.3–5.6]), *p* < 0.001. It was also statistically significantly positively correlated with PIV (r_s_ = 0.268, *p* = 0.007). Red cell distribution width is a parameter easily obtained from CBC that shows the size of circulatory erythrocytes in the peripheral blood, and it has been suggested to be an accurate marker of inflammation [39]. Increased RDW reflecting the abnormal erythropoiesis and red blood cell survival was shown to be associated with mortality in various diseases [40,41]. Moreover, serum albumin level is an important marker of nutritional status and a negative acute phase reactant [39]. Recent studies evaluated the prognostic significance of RDW to albumin ratio in aortic aneurysms, coronary artery disease, diabetes mellitus, cancer, stroke and other inflammatory diseases [40,42,43,44,45]. Studies demonstrated that higher RDW/albumin ratio was associated with higher mortality rate [39,44,45]. Our findings support this, and a previous study of 934 burn patients with a post-operative 90-day mortality rate of 22.5%, that demonstrated a significant correlation between the RDW/albumin ratio on postoperative day 1 and 90-day mortality [15].

Several limitations of this study should be acknowledged. The primary limitation of this study was its retrospective, single-center design, which may limit the generalizability of the findings, as it reflects the specific patient demographics, burn characteristics, and clinical management practices of a single institution. Additionally, potentially influential variables—such as nutritional status, socio-economic factors, and subtle variations in treatment—were not captured, and as a tertiary referral hospital, our cohort may include a disproportionately higher number of severe cases, further constraining external validity. A second limitation is that CBC measurements, and thus the PIV, were obtained only at admission. Given the dynamic nature of immune responses and changes in blood cell counts in severe burns, a single measurement may not capture subsequent fluctuations related to tissue injury, infection, or treatment, potentially underestimating PIV’s prognostic performance. Serial monitoring could better reflect temporal trends, delay immune activation, and improve its predictive accuracy for in-hospital mortality. Future prospective, multicenter studies incorporating longitudinal CBC and additional inflammatory markers are warranted to validate these findings and provide a more comprehensive understanding of the relationship between immune dysregulation and mortality in patients with extensive burns. Yet, consistent with our hypothesis, we demonstrated an association between higher PIV at admission and increased mortality in severely burned patients. To the best of our knowledge, this is the first study to evaluate this newly described index in patients with major burns. Third, although infections are a major cause of mortality in patients with extensive burns and can influence immune cell counts, other markers of infection or inflammation, such as CRP and procalcitonin, were not available for all patients and therefore could not be included in the analysis. Nonetheless, all patients were admitted within the first 24 h after burn injury, which reduces the likelihood of pre-existing infection affecting the initial immune cell measurements. The last limitation was as we focused on predicting in-hospital mortality, patients did not have longer follow-up, thus the association between PIV and long-term mortality was not evaluated. Prospective studies with larger patient numbers are required to make a definite conclusion on the predictive value of PIV in both in-hospital and long-term mortality in patients with severe burn.

## 5. Conclusions

The findings of this study showed that indices derived from CBC which is routinely used in daily practice may help to predict the in-hospital mortality of patients with extensive burn. The recently defined comprehensive index PIV was proven to be a reliable immuno-inflammatory marker for its prognostic accuracy in severe burn patients for the first time by this study. Early detection of mortality risk in this devastating injury may allow for timely implementation of tailored interventions and more intensive treatments at critical stages, ultimately improving patient survival rates while alleviating the significant economic burden on healthcare systems associated with severe burns.

## Figures and Tables

**Table 1:** The demographic, clinical and laboratory characteristics of patients with extensive burn including survivors and non-survivors.

	All Patients (*n* = 100)	Survivors (*n* = 77)	Non-Survivors (*n* = 23)	*p*-Value
Sex, *n* (M/F)	79/21	62/15	17/6	0.56 ^a^
Age (years) ^+^	41 (26.3–55)	41 (26–54.5)	43 (29–61)	0.43 ^b^
Total burn surface area ^+^	27.5 (20–50)	24 (20–35)	60 (40–75)	**<0.001**
Surgical therapy *	81 (81)	66 (85.7)	15 (65.2)	**0.037** ^a^
R-Baux ^+^	76 (59.3–103.8)	67 (55–87.5)	113 (91–135)	**<0.001** ^b^
Abbreviated Burn Severity Index^+^	6 (5–9)	6 (5–7)	11 (8–12)	**<0.001**
BOBI	2 (1–4)	2 (1–4)	4 (2–6)	**0.017** ^b^
CTBS	4 (3–5)	4 (3–5)	5 (3–8)	**0.021** ^b^
SOFA	1 (0–3)	0 (0–3)	1 (0–4)	0.10 ^b^
Etiology of burn *				0.089 ^c^
Flame	69 (69)	49 (63.6)	20 (82.6)	
Electrical	8 (8)	8 (10.4)	0 (0)	
Scald	22 (22)	19 (24.7)	3 (13)	
Chemical	1 (1)	1 (1.3)	0 (0)	
Inhalation injury *	27 (27)	10 (13)	17 (73.9)	**<0.001** ^c^
Comorbidities *				
Hypertension	14 (14)	9 (11.7)	5 (21.7)	0.30 ^a^
Diabetes mellitus	6 (6)	5 (6.5)	1 (4.3)	>0.99 ^a^
CHD	5 (5)	2 (2.6)	3 (13)	0.078 ^a^
Laboratory Features ^+^				
Albumin	3.1 (2.1–3.8)	3.4 (2.4–4)	2 (1.7–2.4)	**<0.001**
Hb	14.4 ± 3.1	14.3 ± 2.7	14.8 ± 4.4	0.59 ^d^
MCV	87.9 ± 5.1	88 ± 4.7	87.4 ± 6.4	0.59 ^d^
MPV	8.7 (7.9–9.4)	8.6 (7.9–9.5)	8.7 (8.3–9.2)	0.40 ^b^
RDW	13.5 (13–14.1)	13.3 (12.9–13.9)	13.8 (13.3–15.3)	0.18 ^b^
Neutrophils (×10^9^ L)	9.28 (7.01–13.95)	8.5 (6.62–12.21)	14.9 (10.6–21.4)	**<0.001** ^b^
Lymphocytes (×10^9^ L)	1.5 (1.1–2.19)	1.5 (1.15–2.49)	1.3 (0.9–1.9)	0.16 ^b^
Monocytes (×10^9^ L)	0.9 (0.6–1.26)	0.87 (0.59–1.2)	1.1 (0.7–1.9)	0.064 ^b^
Platelets (×10^9^ L)	214.5 (175–287)	233 (175.5–290)	189 (138–228)	0.070 ^b^
LPR	7.39 (5.1–10.65)	7.37 (5.13–10.64)	8.15 (3.98–12.81)	0.90 ^b^
NLR	6.13 (3.3–10.74)	5.29 (2.79–8.46)	10.32 (7.39–18.67)	**<0.001** ^b^
MLR	56.7 (30.7–94.17)	47.93 (29.28–84.03)	76.92 (58.33–111.11)	**0.012** ^b^
RDW/Albumin	4.4 (3.5–6.4)	3.9 (3.3–5.6)	6.4 (5.7–5.2)	<0.001
SII	2.9 (1.27–5.38)	2.33 (1.16–4.48)	4.82 (3.86–9.48)	**<0.001** ^b^
SIRI	4.76 (2.19–12.87)	4.31 (1.81–8.11)	11.61 (5.17–28.88)	**<0.001** ^b^
PIV	1043.1 (464–2430.1)	978.4 (418–1530.2)	1568 (842.4–5189.4)	**0.009** ^b^
PIV *				0.002 ^c^
<1185	58 (58)	51 (66.2)	7 (30.4)	
≥1185	42 (42)	26 (33.8)	16 (69.6)	

R-Baux; revised Baux, BOBI; Belgian Outcome in Burn Injury, CTBS; Cape Town Burn Score, CHD; chronic heart disease, Hb; hemoglobin, RDW; red cell distribution width, MPV; mean platelet volume, MCV; mean corpuscular volume, LPR; lymphocyte/platelet ratio, MLR; monocyte to lymphocyte ratio, NLR; neutrophil/lymphocyte ratio, NMR; Neutrophil-to-Monocyte Ratio, NLPR; Neutrophil-to-Monocyte-to-Platelet Ratio, SOFA; Sepsis-related Organ Failure Assessment, SII; systemic immune inflammatory index, SIRI; systemic inflammation response index, HALP; Hemoglobin Albumin Lymphocyte Platelet Score, PIV; Pan-immune inflammation value. * *n* (%), ^+^ median (IQR, 25–75 percentiles), ^a^ Fisher’s Exact Test, ^b^ Mann–Whitney U, ^c^ Chi-Square, ^d^ Independent-Samples T Test. The items written in bold are statistically significant.

**Table 2:** The correlation of Pan-immune inflammation value with other inflammatory indices and burn severity scores.

	PIV	
	r_s_	*p*-Value
Lymphocte/platelet ratio	−0.385	**<0.001**
Neutrophil/lymphocyte ratio	0.747	**<0.001**
Monocyte/lymphocyte ratio	0.800	**<0.001**
Red Cell Distrubution Width/Albumin	0.268	**0.007**
Systemic Immun-Inflammatory Index	0.602	**<0.001**
Systemic Inflammatory Response Index	0.910	**<0.001**
Revised Baux Score	0.169	0.093
Abbreviated Burn Severity Index	0.229	**0.022**
Belgian Outcome in Burn Injury	0.108	0.29
Cape Town Burn Score	0.068	0.50
SOFA Score	0.117	0.25

SOFA; Sepsis-related Organ Failure Assessment. The items written in bold are statistically significant.

**Table 3:** Predictive performance of mortality scoring systems based on the area under the receiver operating characteristic analysis, sensitivity, specificity, positive predictive value, and negative predictive value.

	AUC	Cut-Off	Sensitivity (%)	Specificity (%)	PPV (%)	NPV (%)
ABSI	0.90	8	91.3	76.6	53.8	96.7
r-Baux	0.83	67	100	51.9	38.3	100
NLR	0.74	7.4	78.3	70.1	43.9	91.5
PIV	0.68	1185	69.6	66.2	38.1	87.9
BOBI	0.66	3	65.2	61	33.3	85.5
CTBS	0.66	4	73.9	48.1	29.8	86
SOFA	0.61	1	65.2	53.2	29.4	83.7

ABSI; Abbreviated Burn Severity Index, AUC; Area under the curve, rBaux; revised Baux, BOBI; Belgian Outcome in Burn Injury, CTBS; Cape Town Burn Score, SOFA; Sepsis-related Organ Failure Assessment, NLR; neutrophil/lymphocyte ratio, NPV; Negative Predictive Value, PIV; Pan-immune inflammation value, PPV; Positive Predictive Value.

**Table 4:** Univariable and Multivariable Regression Analysis of Parameters Associated with Mortality in Patients with Extensive Burn.

	Univariate Analysis		Multivariate Analysis	
	OR (95% CI)	*p* Value	OR (95% CI)	*p* Value
Surgical therapy	3.20 (1.10–9.32)	0.033	11.53 (1.45–91.85)	**0.021**
Inhalation injury	18.98 (6.05–59.57)	<0.001	10.93 (2.43–49.11)	**0.002**
Albumin	0.26 (0.13–0.51)	<0.001	0.40 (0.15–1.06)	0.064
PIV (≥1185 → <1185)	4.48 (1.64–12.26)	0.003	5.06 (1.03–24.76)	**0.045**

PIV: Pan-immune inflammation value. The items written in bold are statistically significant.

## Data Availability

The original contributions presented in this study are included in the article; further inquiries can be directed to the corresponding authors.

## Abbreviations

The following abbreviations are used in this manuscript:

PIV	Pan-immune inflammation value
ROC	Receiver operating characteristic
TBSA	Total body surface area affected by the burn
NLR	Neutrophil/lymphocyte ratio
LPR	Lymphocyte/platelet ratio
MLR	Monocyte to lymphocyte ratio
NLPR	Neutrophil-to-Monocyte-to-Platelet Ratio
SII	Systemic immune inflammatory index
SIRI	Systemic inflammation response index
HALP	Hemoglobin Albumin Lymphocyte Platelet Score
CBC	Complete blood count
Hg	Hemoglobin
RDW	Red cell distribution width
MPV	Mean platelet volume
MCV	Mean corpuscular volume
ABSI	Abbreviated Burn Severity Index
rBaux	The Revised Baux Score
SPSS	Statistical Package for the Social Sciences
CHD	Chronic heart disease
STEMI	ST-segment elevation myocardial infarction
